# Translational Neurodegeneration, a platform to share knowledge and experience in translational study of neurodegenerative diseases

**DOI:** 10.1186/2047-9158-1-1

**Published:** 2012-01-13

**Authors:** Shengdi Chen, Jialin C Zheng

**Affiliations:** 1 Department of Neurology & Institute of Neurology, Rui Jin Hospital, Shanghai Jiao Tong University School of Medicine, Shanghai, China; 2 Department of Pharmacology and Experimental Neuroscience, University of Nebraska Medical Center, Omaha, NE, USA

## 

A common feature of current biomedical science is inter-disciplinary research and collaboration. Translational research, the basis for translational medicine, integrates the basic sciences and clinical medicine with the aim of optimizing preventive measures and patient care, is at the cusp for expansion. Translational medicine, in short, is the process of turning appropriate biological discoveries into drugs and medical devices that can be used in the treatment of patients. Vigorous efforts have been made to link basic scientific research with clinical investigations. Notably, in the area of neurodegenerative disorders, more basic and clinical researchers as well as nations have joined forces to explore the interface between basic neurosciences and clinical neurology and psychology. Thus, the newly established journal of *Translational Neurodegeneration *will provide a perfect platform at the global level to share knowledge and experience of the latest research on the epidemiology, etiology, pathogenesis, diagnosis, management and prevention of neurodegenerative diseases.

Indeed, the prevalence of neurodegenerative disorders, including Alzheimer's disease (AD) and Parkinson's disease (PD), has increased significantly as global populations age. Specifically, the number of cases of dementia in the developed world is projected to rise from 13.5 million in 2000 to 21.2 million in 2025, and to 36.7 million in 2050[1]. Currently, the number of deaths caused by AD is only next to the number of deaths caused by stroke. As the prevalence of AD grows, so does the cost to a nation. For PD, the second most common neurodegenerative disease after AD, more than 4 million people suffer from this devastating disease worldwide and that will double in the next 25 years [2]. To date, PD is still an incurable progressive neurological disorder that seriously impairs the quality of life.

The discovery and application of levodopa (L-dopa) is one of the best examples of translational research for neurodegenerative diseases. In 1910s, L-dopa was first isolated from seedlings of Vicia faba; and in 1938, L-dopa decarboxylase was discovered, which can produce dopamine (DA) from L-dopa. In 1959, DA was found enriched in the basal ganglia; and in 1960, a severe striatal DA deficit was demonstrated in PD patients. These major discoveries and a deepening understanding of the neurochemistry of DA and the neuropathology of PD led to the concept of "DA replacement" with L-dopa. In 1961, L-dopa was tried in PD patients by i.v. treatment. In 1967, oral administration of L-dopa was reported to produce dramatic improvements in PD patients with increasing amounts over long periods [3]. However, the main side effects of increasing L-dopa administration, i.e., dyskinesias and motor fluctuations, became apparent. This clinical finding confused doctors and patients, and a solution was needed. In 1970s, the key cause was found. L-dopa decarboxylase degraded L-dopa to DA in peripheral blood, which can not across the blood-brain barrier. These findings led to the first L-dopa combination, benserazide/L-dopa or carbidopa/L-dopa by adding a dopa decarboxylase inhibitor to treatment, which showed advantages in reducing side effects and gaining better symptom control [4]. Since then, PD researchers have attempted to overcome complications with such techniques as continuous L-dopa infusion and, most recently, long-acting L-dopa combinations.

Substantial progress has been made in understanding the pathogenesis of neurodegenerative diseases, but we are still a long way from applying it to clinical application. Translational research will definitely help overcome the bottleneck and barriers in 1) identifing and validating biomarkers for early or pre-clinical diagnosis as well as clinical progression; 2) promoting the innovative clinical technologies, such as neuroimaging, stem cell technology and nanotechnology; and 3) developing the novel drug candidates; and more importantly, to gain a multitude of new ideas and contacts back to research. Thus, launching a journal in translational neurodegenerative diseases is essential and timely.

The latest research on the etiology, pathogenesis, potential diagnostic markers and treatment modalities of PD has been presented at the XIX World Congress on Parkinson's Diseases and Related Disorders. Almost 2500 distinguished scientists, clinicians and allied health experts gathered in Shanghai, China, December 11-14, 2011 to enhance collaboration in these important inter-disciplinary research areas. Organized by the Association of Parkinsonism and Related Disorders and the local host, the Department of Neurology, Ruijin Hospital affiliated to Shanghai Jiao Tong University School of Medicine, the congress is the highest level of academic conferences in the field of PD and related disorders. Several speakers and attendees contributed review and original articles for the inaugural issue of *Translational Neurodegeneration*. Notably, the articles are interdisciplinary and focus on many current issues facing translational neuroscience research.

Research of gene mutation, especially of alpha-synuclein, has provided important clues in understanding the pathogenesis of PD. In cell models, A53T mutation of alpha-synuclein can increase macroautophagy, which has not yet been detected in patients. Yue Huang et al [5]aimed in their article "Macroautophagy in sporadic and the genetic form of Parkinson's disease with the A53T α-synuclein mutation" to confirm the above changes in brain tissue of PD patients. Interestingly, they couldn't find any evidence to support the conclusion that alpha-synuclein is removed by macroautophagy; and the results may be opposite to the previous reports. This is followed by a review, provided by Dr. Shu-Leong Ho's group, titled "Mitochondrial neuronal uncoupling proteins: a target for potential disease-modification in Parkinson's disease" [6]. It gave a brief insight into the role of mitochondrial dysfunction and oxidative stress in the converging pathogenic processes involved in PD and demonstrated that mitochondrial uncoupling proteins (UCPs) may play a vital role in protecting neurons from various pathogenic stresses including those associated with PD. UCPs would be another potential target for the treatment of these neurological disorders.

Peter A. LeWitt discussed the wide role of norepinephrine (NE) in his review titled "Norepinephrine: the next therapeutics frontier for Parkinson's disease" [7]. NE may affect both the motor and non-motor functions, such as cognition, depression, circadian rhythms, and orthostatic hypotension. More importantly, drugs like β-adrenoceptor antagonist and α2-adrenergic receptor antagonists might help to ameliorate motor, cognitive, and affective features of PD, suggesting NE's potential role in the treatment of motor and non-motor symptoms of PD.

Jon Stoessl introduced the latest progress in "Neuroimaging in the early diagnosis of neurodegenerative diseases" [8]. SPECT and PET technologies have been widely used to assess the pre-and post-synaptic abnormalities of transmission to differentiate PD from the Parkinson-plus syndromes. DTI (diffusion tensor imaging), fMRI, task-free fMRI and FDG-PET may also provide help in the differential diagnosis of degenerative dementias. The use of multimodal imaging, especially when combined with other biomarkers, showed increasing promise for the early diagnosis of neurodegenerative diseases.

Translational research and knowledge of neurodegeneration are expanding at a fast pace. The potential to find causes, treatments and cures for many neurological and neurodegenerative diseases are available to us. This congress and our journal highlight the opportunities for interdisciplinary and global collaborations. By offering a high-visibility forum for new insights and discussions of translational research in neurodegenerative diseases, *Translational Neurodegeneration *will promote the rapid conversion of basic research to clinical applications benefiting patients better and faster. The concept and spirit of translational research in the field of neurodegenerative disorders will spread through *Translational Neurodegeneration *and the authors.

We thank the authors, referees, and Springer and BioMed Central, our publisher, for their help in making this journal and first issue possible. Special thanks also go to Ms. Robin Taylor from the University of Nebraska Medical Center; Drs. Jianqing Ding and Yuyan Tan, managing Editors of *Translational Neurodegeneration*; Rui Jin Hospital leaders, colleagues, team members and leaders of Shanghai Jiao Tong University School of Medicine, University of Nebraska Medical Center, Society for Neurology Chinese Medical Association and Chinese Society for Neuroscience, without whose help the success of this undertaking could not have been realized.
